# Production of Phytotoxic Cationic α-Helical Antimicrobial Peptides in Plant Cells Using Inducible Promoters

**DOI:** 10.1371/journal.pone.0109990

**Published:** 2014-11-11

**Authors:** Nuri Company, Anna Nadal, Cristina Ruiz, Maria Pla

**Affiliations:** Institute for Food and Agricultural Technology, University of Girona, Girona, Spain; Nanjing Agricultural University, China

## Abstract

Synthetic linear antimicrobial peptides with cationic α-helical structures, such as BP100, have potent and specific activities against economically important plant pathogenic bacteria. They are also recognized as valuable therapeutics and preservatives. However, highly active BP100 derivatives are often phytotoxic when expressed at high levels as recombinant peptides in plants. Here we demonstrate that production of recombinant phytotoxic peptides in transgenic plants is possible by strictly limiting transgene expression to certain tissues and conditions, and specifically that minimization of this expression during transformation and regeneration of transgenic plants is essential to obtain viable plant biofactories. On the basis of whole-genome transcriptomic data available online, we identified the *Os.hsp82* promoter that fulfilled this requirement and was highly induced in response to heat shock. Using this strategy, we generated transgenic rice lines producing moderate yields of severely phytotoxic BP100 derivatives on exposure to high temperature. In addition, a threshold for gene expression in selected tissues and stages was experimentally established, below which the corresponding promoters should be suitable for driving the expression of recombinant phytotoxic proteins in genetically modified plants. In view of the growing transcriptomics data available, this approach is of interest to assist promoter selection for specific purposes.

## Introduction

Antimicrobial peptides (AMPs) are key components of innate immunity in plants and animals, and are also produced by microbes in antibiosis processes. A significant proportion are strongly cationic and have linear structures that adopt an amphipathic α-helical conformation that binds to the phospholipid membranes of target microbes before the hydrophobic face is inserted into the membrane bilayer. This unique mode of action explains the lack of resistance in target pathogens, and makes AMPs valuable novel therapeutic agents against bacteria, fungi, viruses, parasites and tumor cells. Improved or synthetic AMPs have been designed with increased potency against selected pathogens [1]–[5]. As an example, the synthetic undecapeptide BP100 (KKLFKKILKYL-NH_2_) is effective against *Xanthomonas vesicatoria* in pepper, *Erwinia amylovora* in apple and *Pseudomonas syringae* in pear [3] with the same efficacy as standard antibiotics, while being biocompatible, as determined by acute oral toxicity tests in mice [6]. Plant expression of recombinant cationic α-helical peptides such as BP100 is preferable for industrial and phytosanitary applications. We have recently showed that active BP100-derived peptides can be expressed as recombinant peptides in plants [7], [8], demonstrated by the increased resistance of GM plants to some rice pathogens [7] and by *in vitro* growth inhibition assays [8]. These recombinant BP100-derived peptides include endoplasmic reticulum (ER) retention motifs to minimize toxicity to the host plant. This did not affect the antimicrobial activity of the products *in vitro*, demonstrated using bacterial growth inhibition tests. Following transient expression in *Nicotiana benthamiana* and stable expression in *Arabidopsis thaliana* seedlings, the peptides accumulated in large ER-derived vesicles, along with typical ER luminal proteins [8]. However, the authors found that the recombinant peptides were often toxic to the host during later developmental stages. Similarly, most transgenic *Oryza sativa* plants constitutively expressing recombinant ER-targeted BP100-derived peptides failed to achieve maturity, with only a few peptides coupling potent antimicrobial and low hemolytic activity accumulating in transgenic rice lines, with a yield of up to 0.5% total soluble protein (TSP) [8]. Not many cationic α-helical peptides can be expressed in transgenic plants following this strategy, although they have potent activities against other types of pathogenic cells making them valuable as novel therapeutics and preservatives.

High temperature stress is one of the most common abiotic stresses among many world crops. Plants have evolved various physiological and molecular mechanisms to resist heat stress. Based on the expression data from different plant species, it has been estimated that high temperatures affect approximately 2% of the plant genome (review in [9]). Exhaustive identification of heat stress-responsive genes has been carried out by means of transcriptomics [10]–[15]. Two groups of genes have been found, signaling components (e.g. protein kinases and transcription factors) and functional genes such as heat shock proteins (Hsps) [16], although many heat shock genes still have unknown functions. Hsps are functionally linked to molecular chaperones that are essential for maintenance and restoration of protein homeostasis. Protein denaturation occurring during stress triggers high transcription of *hsp* genes by the binding of active heat shock factors (Hsfs) to heat shock elements. Alterations in expression of a high number of genes in response to stress occur in complex systems. Cross-talk seems to exist between the regulatory pathways in response to different abiotic stresses such as temperature, and osmotic or oxidative, and biotic stresses [17]. On fusing several heat-shock promoters to reporter genes, they have been found to be regulated during various stress situations, have organ and developmental stage-specific basal expression levels and are induced by stress [18].

Heat-shock induced genes can be highly up-regulated at the transcriptional level by exposure to temperatures above those of normal plant growth. Therefore, induction can be easily carried out, is inexpensive and does not require the use of hormones or chemicals. Here we explored the use of heat-shock inducible promoters with minimal basal activity during normal plant development, to allow expression of recombinant phytotoxic peptides in transgenic rice. Rice has emerged as a powerful platform for large-scale production of recombinant proteins; it has easy cropping conditions and is a self-pollinating crop [19], [20]. It is a most suitable crop for genetic manipulation due to its small genome size and the well-established gene transfer technology. Moreover, the availability of the rice complete genome sequence, the exponential growth of profiling studies reporting gene expression data for rice, and the development of databases and bioinformatics tools, provide a possible way to identify genes related to heat stress responses, and with specific expression patterns.

## Materials and Methods

### *In silico* identification of candidate heat shock promoters

Miamexpress-EMBL-EBI (http://www.ebi.ac.uk/miamexpress/) and GEO-NCBI (http://nlmcatalog.nlm.nih.gov/geo/) were used to identify published microarray experiments questioning the late response of rice seedlings to heat shock stress. In the experiment with accession number GSE14275, the authors [10] state that rice Affymetrix microarrays were hybridized with RNA extracted from 14-day-old rice seedlings grown in a growth chamber with a daily photoperiodic cycle of 14 h light and 10 h dark, at between 28–30°C, with or without being subjected to 42 °C for 3 h. The data from this experiment was extracted using the RMA software [21], which includes background adjustment, quantile normalization and summarization, and used to identify probes for subsequent analysis.

Rice gene expression data in different organ and developmental stages, and in response to various stress conditions, was obtained from the CREP database (Collection of Rice Expression Profiles; http://crep.ncpgr.cn/crep-cgi/home.pl) and the microarray hybridization results available at the Miamexpress-EMBL-EBI and GEO-NCBI websites.

The NetAffx-analysis center (www.affymetrix.com) allowed identification of the gene associated to every probe, and the sequence was retrieved from GenBank (http://www.ncbi.nlm.nih.gov/genbank). Likely promoter sequences were identified using the PlantProm DB, an annotated and non-redundant database of proximal promoter sequences [22], [23].

### Primer design and amplification of rice promoter sequences

Primer blast (http://www.ncbi.nlm.nih.gov/tools/primer-blast/) was used to design primer pairs specifically targeting the identified promoter sequences, blocking the reverse primer at the −1 position (immediately upstream of the ATG translation start codon). Two primer pairs (Table S1) were designed to amplify 553 and 1016 bp fragments of the pHsp18 and pHsp82 promoters. They each included an enzyme restriction site (*Kpn*I and *Spe*I at the distal and proximal promoter end, respectively) to facilitate the subsequent cloning steps.

*Oryza sativa* L. ssp. *japonica*, v. Senia was grown under controlled conditions at 28±1°C and with a 16 h light / 8 h dark photoperiod with fluorescent Sylvania Cool White lamps. Genomic DNA was extracted from 1 g of leaf samples, obtained from young plants, using the commercial NucleoSpin Plant II kit (Macherey-Nagel, Düren, Germany) according to the manufacturer's instructions.

PCR was in a final volume of 50 µl 1x reaction buffer with the appropriate concentrations of MgCl_2_ and primers (Fisher Scientific SL, Madrid, Spain) (Table S1), 200 µM dNTPs and 1 unit Expand High Fidelity DNA polymerase (Roche Diagnostics GmbH, Mannheim, Germany). The reaction conditions were as follows: 3 min at 94°C; 10 cycles of 15 s at 94°C, 30 s at the appropriate annealing temperature (Table S1) and 1 min at 72°C; 20 cycles of 15 s at 94°C, 30 s at the same annealing temperature and 1 min, plus an additional 5 s for each successive cycle, at 72°C; and a final extension of 10 min at 72°C.

### Construction of plant transformation vectors

The pHsp18 and pHsp82 promoters were separately subcloned into the *Kpn*I and *Spe*I sites of a pBluescriptIIKS + derived vector having a polylinker fragment with the *Kpn*I, *Spe*I and *BamH*I restriction sites, followed by the *A. tumefaciens* nopaline synthase *nos* terminator sequence. The sequences encoding BP100.2 [7], BP100-DsRed-tag54 and DsRed-tag54 [8] were subcloned into these plasmids using the *Spe*I and *BamH*I restriction sites.

For the BP100.2 plasmids, the sequence encoding the *N. tabacum* pathogenesis related protein PR1a signal peptide [24] fused to BP100.2 [7] was PCR amplified using pAHC17-bp100.2 [7] as template and the primers PR1a_Spe and BP100KDEL_Bam, with the *Spe*I and *BamH*I restriction sequences respectively. For DsRed-tag54 control plasmids, the sequence encoding the *Petrosilinum hortense* chalcone synthase 5′ untranslated region plus the codon-optimized leader peptide derived from the heavy chain of the murine mAb24 monoclonal antibody [25] fused to DsRed [26], the epitope tag54 [27] and the KDEL ER retention motif, were PCR amplified using pCdsred-tag54 [8] as template and the primers CHS_Spe and TagKDEL_Bam, that included the required restriction sites. As an additional control, a BP100-DsRed-tag54 plasmid was generated similarly to DsRed-tag54, and including the BP100 antimicrobial sequence placed N-terminal to DsRed-tag54. The coding sequence was obtained by amplification of the pCbp100-dsred-tag54 DNA [8] with the same CHS_Spe and TagKDEL_Bam primers. PCR was in a final volume of 50 µl 1x reaction buffer with the appropriate concentrations of MgCl_2_ and primers (Fisher Scientific SL, Madrid) (Table S1), 200 µM dNTPs and 1 unit Expand High Fidelity DNA polymerase (Roche Diagnostics Corporation). Reaction conditions were as follows: 3 min at 94°C; 10 cycles of 15 s at 94°C, 30 s at 49°C and 1 min at 72°C; 20 cycles of 15 s at 94°C, 30 s at 58°C and 1 min, plus additional 5 s each successive cycle, at 72°C; and a final extension of 10 min at 72°C.

On sequence verification (Macrogen, Seoul, Korea) using the CLCbio software (Aarhus, Denmark), the complete constructs, *bp100.2*, *bp100-dsred-tag54* and *dsred-tag54* plus promoter and terminator elements, were directionally inserted into the *Kpn*I and *Sbf*I sites of pCAMBIA1300. The resulting binary vectors were transferred into *A. tumefaciens* strain EHA105 by cold shock [28].

### Production of transgenic rice plants

Commercial *japonica* rice (*Oryza sativa* L.) var. Senia was transformed by *A. tumefaciens* to obtain transgenic rice lines expressing the chimeric proteins mentioned above, using hygromycin resistance as the selection trait. The control plasmid, pCambia1300 (transferring only the *hpt*II selection gene), was transformed in parallel. Embryonic calluses derived from mature embryos were transformed as previously described [29]. Hygromycin resistant T_0_ plants were grown to maturity under standard greenhouse conditions to obtain the T_1_ generation. Leaf samples of individual T_0_ plants at the 5-leaf vegetative stage were used to extract genomic DNA and assess the presence and copy number of the transgene by real-time PCR (qPCR), targeting the coding region of every transgene as previously described [8]. The *actin* endogenous gene was used to normalize the Ct values. The number of fertile genetically modified (GM) plants containing every plasmid was recorded to calculate the transformation efficiency compared to that of the control pCambia 1300 plasmid (number of fertile GM plants obtained per initial callus. This value was then normalized with that obtained for the control plasmid).

### Analysis of gene expression: plant material, RNA isolation and RT-PCR

#### Plant material: heat shock treatment

Wild type rice seeds (var. Senia) were surface sterilized and germinated *in vitro* in sterile MS medium [30], including vitamins (2.2 g/L MS medium, 8 g/L agar and 30 g/L sucrose), under controlled conditions (28±1°C temperature and a 16 h light / 8 h dark photoperiod with fluorescent Sylvania Cool White lamps). After one week (two-leaf vegetative stage, V2), groups of 15 plants were treated at 42°C for 0, 1, 2, 4, 8 and 16 h. For each treatment, three groups of five plants were collected as biological replicates, immediately frozen in liquid nitrogen and stored at −80°C.

For each GM event, 15 T_1_ seeds were surface sterilized and *in vitro* cultured, as mentioned above, up to the V2 stage. They were treated at 42°C for 0 and 2 h and their leaves individually frozen in liquid nitrogen. The presence of the transgene was individually assessed in every plant using the Phire Plant Direct PCR Kit (Thermo Scientific, Lithuania) combined with the DsRed_for and Nos.te_rev (constructs with *dsred-tag54* and *bp100-dsred-tag54*) or the P82_for and Nos.te_rev (constructs with *bp100.2*) primer pairs (Table S1), according to the manufacturer's instructions. Samples giving a positive PCR signal (i.e. harboring the transgene) were mixed for subsequent analyses.

#### Plant material: monitoring of the transformation process

The transformation to obtain transgenic rice was monitored using *A. tumefaciens* strain EHA105, with or without the basic pCambia1300 vector (with *hpt*II). Embryonic calluses derived from rice mature embryos were transformed and, when *hptII* was transformed, selected with hygromycin [29]. Samples of ten independent calluses or events were taken at eight different stages for each transformation.

For embryo extraction, 120 hygromycin-resistant seeds (harboring *hptII*) and 80 non-transformed seeds were surface-sterilized and their embryos were immediately extracted and frozen, or germinated in 500 µL water or 500 µL water with 45 mg/L hygromycin B (40 seeds per treatment and embryo type). Germination was in a culture chamber (28±1°C with a 16 h light/8 h dark photoperiod, under fluorescent Sylvania Cool White lamps) for two and five days prior to embryo extraction and immediate freezing in liquid nitrogen. Untransformed seeds were not treated with hygromycin B.

#### RNA isolation and reverse transcription (RT) coupled to qPCR

Samples were homogenized in liquid nitrogen and 100 mg was used to extract RNA with the Trizol reagent (Invitrogen, Karlsruhe, Germany) based protocol. The DNase I digestion (Ambion, Grand Island, NY) was carried out according to the manufacturer's protocol, and RNA concentration and quality was assessed by UV absorption at 260 and 280 nm using a NanoDrop ND1000 spectrophotometer (Nanodrop technologies, Wilmington, DE). RT-qPCR was carried out as previously described [7]. For each sample, cDNA was synthesized with random primers in duplicate and qPCR reactions targeting *bp100.2* and *dsred-tag54* transgenes, and *Os.hsp18* and *Os.hsp82* rice genes were carried out in triplicate. The qPCRs were in a final volume of 20 µl containing 1X SYBR Green PCR Master Mix (Applied Biosystems, Foster City, CA, USA), the appropriate concentrations of primers (Fisher Scientific SL, Madrid, Spain) (Table S1) and 1 µl cDNA. The reaction conditions were as follows: 10 min at 95°C for initial denaturation; 50 cycles of 15 s at 95°C and 1 min at 60°C; and a final melting curve program of 60 to 95°C with a heating rate of 0.5°C/s. Melting curve analyses produced single peaks, with no primer-dimer peaks or artifacts, indicating the reactions were specific. All reactions had a linearity coefficient exceeding 0.995 and efficiency values above 0.95. The *ef1α* gene was used for normalization, its suitability having been confirmed using the geNORM v3.4 statistical algorithm ([31]; *M* values below 0.5 in our samples). Triplicate biological samples, each containing at least five plants, were analyzed in each case.

### Protein extraction and western blot analyses

For each GM event, 15 T_1_ seeds were surface sterilized and *in vitro* cultured as mentioned above, up to the V2 stage. Rice seedlings were treated at 42°C for 2 h, then transferred to 28±1°C with a 16 h light/8 h dark photoperiod under fluorescent Sylvania Cool White lamps for two days, to allow recombinant protein accumulation and DsRed maturation, and then immediately frozen in liquid nitrogen. The Phire Plant Direct PCR Kit (Thermo Scientific, Lithuania) was used in combination with the SYDsRed_for and SYDsRed_rev primers (Table S1), according to the manufacturer's instructions, to individually identify transgenic T_1_ plants. Transgenic seedlings were homogenized in liquid nitrogen and TSP extracted as previously described [8]. Insoluble proteins in the samples expressing *bp100-dsred-tag54* were re-extracted by boiling for 10 min in the same buffer supplemented with 8 M urea and 1% SDS. Protein concentration was determined using the Sigma Bradford Reagent and bovine serum albumin as standard. Twenty µg TSP were separated by PAGE, using 18% (w/v) SDS polyacrylamide gels, and electrotransferred to nitrocellulose membranes. Twenty-five to 1 pmol chemically synthesized controltag54 (GQNIRDGIIKAGPAVAVVGQATQIAKAGPAKDWEHLKDEL), mixed with 20 µg TSP extracted from untransformed rice seedlings, were included to allow quantification of the recombinant peptides. They were hybridized overnight with the mAb54k monoclonal antibody [27] (1∶1,500 dilution) at 4°C, and with horseradish peroxidase-conjugated anti-mouse IgG as the secondary antibody (GE Healthcare Life sciences) (1∶10,000 dilution) for 1 h at room temperature. The hybridization signal was detected by ECL chemiluminescence (Luminata Forte HRP Chemiluminescence Detection Reagents, Millipore – Darmstadt, Germany).

### Phenotype evaluation

T1 seeds carrying pHsp82::bp100.2, pHsp82::dsred-tag54 (three independent events per construct) or hptII, and conventional Senia seeds, were surface sterilized and *in vitro* cultured as mentioned above, up to the V2 stage (height, 6±1 cm). They were all treated at 42°C for 2 h and allowed to recover for three days under the same conditions. Plant growth was monitored daily by measuring the height of the aerial part. Transgenic plants were identified using the Phire Plant Direct PCR Kit (Thermo Scientific, Lithuania) as mentioned above, and only those harboring the transgene (at least five plants per event) were included in statistical analyses. As an additional control, five untransformed Senia plants were grown in parallel and not subjected to heat shock.

### Confocal microscopy

Transgenic rice seedlings, obtained as mentioned above, were treated at 42°C for 2 h and transferred to the standard conditions for two days to allow accumulation of the recombinant protein and DsRed maturation. Radicles were observed under an FV1000 Olympus confocal microscope and red fluorescent images collected with 543 nm excitation using a 550–600 nm emission window. The ImageJ software (http://rsb.info.nih.gov/ij/) was used to calculate the number and size of fluorescent spots and quantify the DsRed fluorescence.

## Results

### Rationale of the approach and selection of suitable promoters

With the increasingly abundant transcriptomics analytical tools and datasets available, we used an *in silico* approach to initially select a small number of candidate heat shock promoters. The EMBL-EBI Array Express website was searched for datasets on transcription profiling of the *Oryza sativa* in response to heat shock. A single microarray experiment was found (E-GEOD-14275, [10]) that questioned 14-day old seedlings, grown at 28–30°C, and with or without heat stress at 42°C. Expression data was available for a total of 22,905 sequences. Those with the highest expression levels had fluorescence intensities up to 13,800, and 16,300 units in microarrays hybridized with RNA from control and heat-shock treated seedlings, respectively. Only 15 and 17 sequences had values above 10,000 fluorescence units. Up to 19 sequences, corresponding to 16 genes, had fluorescence intensities more than 5,500 units higher in heat-shock treated than in control seedlings (Table S2). This included three sequences (AK071613.1, AK071240.1 and CB621753) with very high expression values under induction conditions (above 10,000 fluorescence units) and sequences with the greatest changes in expression in response to heat stress treatment (up to 615-fold, AK063751.1). These sequences were initially selected to drive the expression of phytotoxic peptides in rice.

**Figure 1:**
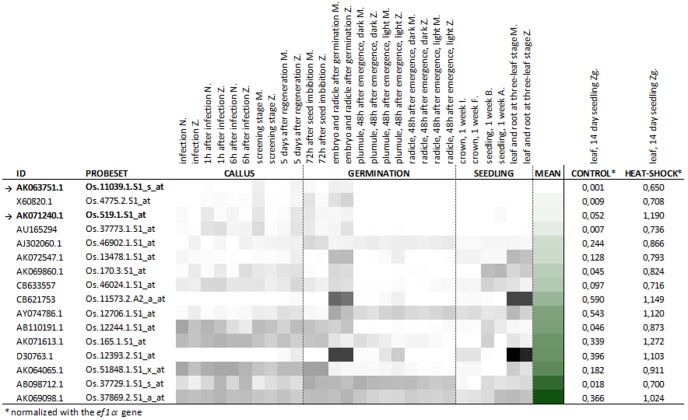
A summary of the expression profiles of a selection of sequences induced by high temperature, in callus and seedlings of eight rice genotypes [Bala (B), FL478 (F), IR29 (I), Minghui 63 (M), Zhenshan 97 (Z), *indica* subspecies, and Azucena (A), Nipponbare (N) and ZhongHua 11 (Zg), *japonica* subspecies], from published *in silico* data. Cluster analysis of 16 genes with the highest expression or induction in response to heat-shock, in a total of 14 tissues considered relevant to the process of obtaining transgenic plants (grey scale). The overall mRNA levels in these tissues and developmental stages were estimated using the mean expression values (green scale). Dark to light color scale represents high to low expression levels, the darkest color corresponding to 90162 (grey scale) and 19788 (green scale) normalized fluorescence units. Arrows indicate the two sequences whose promoters were selected to produce transgenic plants.

The transcription patterns of these 16 genes were subsequently compared with the Collection of Rice Expression Profiles (CREP) database (http://crep.ncpgr.cn/crep-cgi/home.pl), a compilation of quantitative transcriptomes of 39 tissues of various rice genotypes, from the *indica* and *japonica* subspecies, obtained using the commercial Affymetrix Rice GeneChip microarray. We specifically focused on the tissues and developmental stages with special relevance in transformation and regeneration to obtain GM plants. They included callus at different steps of agrotransformation and hygromycin based selection, and imbibed seeds and seedlings at various developmental stages. Multiple gene expression profiles were retrieved using the Multi-genes Chronologer tool (Figure 1). The Os.519.1.S1_at sequence (AK071240.1, encoding a putative 18 kDa Class II Heat Shock Protein) combined low expression levels in all 14 analyzed tissues considered relevant for transformation and very high expression in heat-shock treated seedlings (0.073 and 1.190 normalized fluorescence units). Although it had some expression in control seedlings (0.052 normalized fluorescence units), this promoter was chosen to drive the expression of phytotoxic peptides in plants. A second selected sequence, Os.11039.1.S1_s_at (AK063751.1, encoding a putative Heat Shock Protein 82), had extremely low expression values in the 14 tissues and control seedlings but only moderate expression in response to high temperature treatment (0.033, 0.001 and 0.650 normalized fluorescence units, respectively). There was no induction of Os.519.1.S1_at or Os.11039.1.S1_s_at upon cold, salt or drought stresses, as assessed *in silico* using profiling datasets with GSE6901 as the reference, reporting transcriptome analyses of 7-day old seedlings of Indica rice variety IR64 subjected to 4±1°C, 200 mM NaCl or insufficient moisture in their roots for 3 h (Figure S1).

### Expression patterns of the two selected genes in response to heat shock and during the process of *Agrobacterium tumefaciens* mediated stable transformation of rice using the *hpt*II selection gene

**Figure 2:**
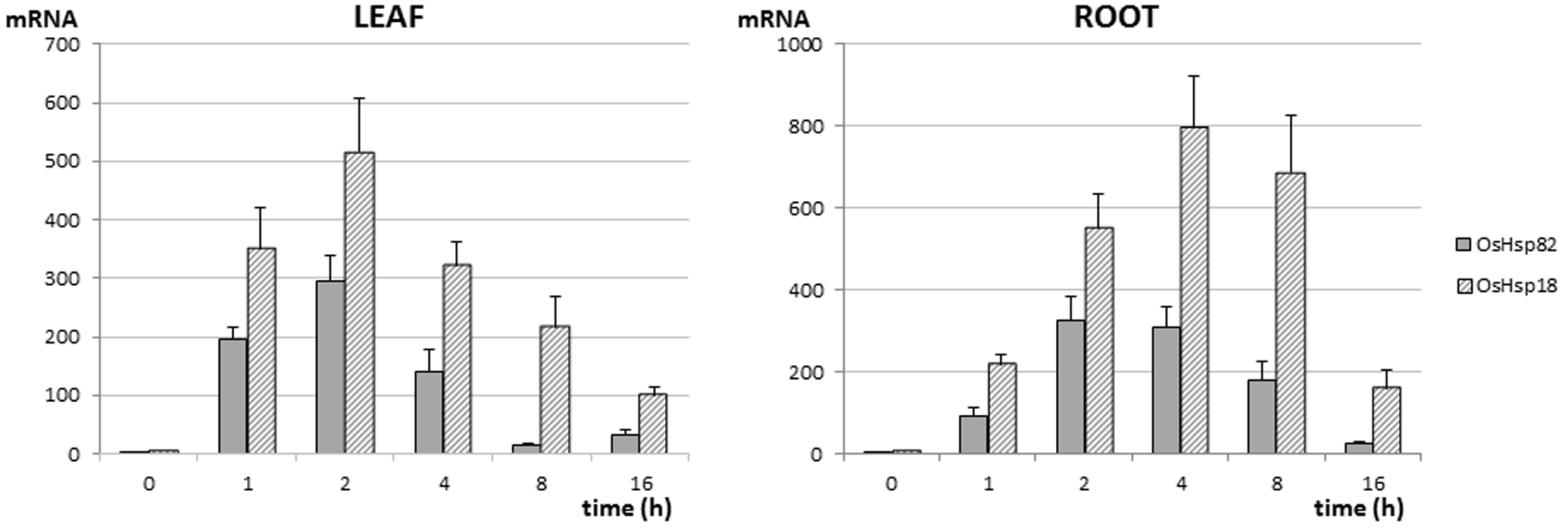
Transcriptional response to high temperature of *Os.hsp18* and *Os.hsp82* in rice var. Senia. Plants at the two leaf vegetative stage were exposed to 42°C for 0, 1, 2, 4, 8 and 16 h, then the mRNA levels of the selected sequences, from leaf and root samples, were analyzed by RT-qPCR. Three biological replicates, each of five plants, with two experimental replicates, were analyzed per treatment. The *ef1*α reference gene (gNORM M value <0.5 in these samples) was used for normalization and the given mRNA values correspond to fold expression vs. the reference gene.

Specific real-time PCR (qPCR) assays were developed to target the coding sequences of *Os.hsp18* and *Os.hsp82* and, coupled to reverse transcription, used to experimentally assess the expression patterns of the selected genes in a range of developmental stages and conditions. Treatment of rice plants with high temperature resulted in increased expression of the two genes (Figure 2). *Os.hsp18* and *Os.hsp82* were highly induced by exposure to 42°C for 2 to 4 h, reaching expression levels of 294 and 516-fold that of *ef1*α in leaves and 326 and 796-fold in roots. There was a decrease in gene expression after these time points.

**Figure 3:**
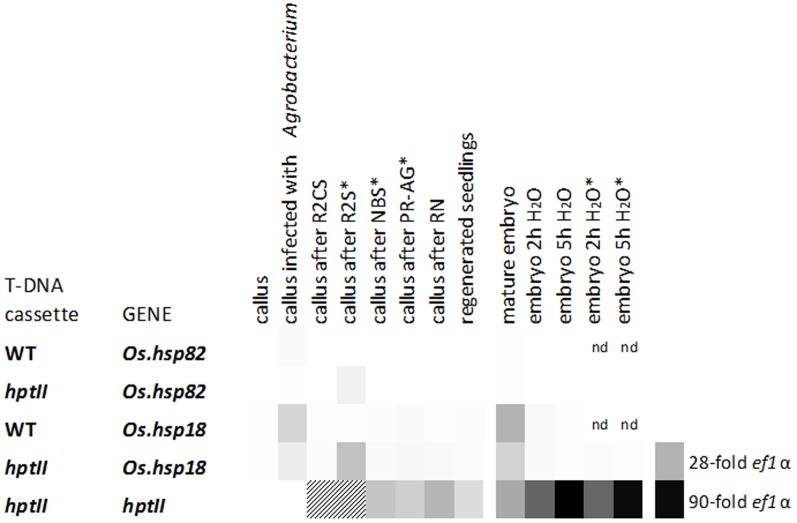
mRNA expression profiles of *Os.hsp18*, *Os.hsp82* and the *hptII* transgene, driven by the CaMV 35S promoter, in rice var. Senia. Different stages during transformation with *A. tumefaciens*, either carrying pCAMBIA1300 (*hptII*) or no T-DNA (WT), and expression in mature and imbibed embryos, are shown. The following stages of callus are shown: 6 weeks after induction of embryogenic callus; immediately after 15 min infection with *A. tumefaciens*; after a 3-day incubation in R2CS medium; after a 2-week incubation in R2S selection medium; 3 weeks after transfer to NBS selection medium; after an 8 to 10-day incubation in PR-AG selection medium; 3–4 weeks after induction of regeneration in RN medium; and two weeks after seedling culture in P medium. Embryos extracted from mature seeds, either before or after imbibition in water for two or five hours, were also analyzed. When the *hptII* DNA cassette was transformed, transgenic cells were selected using hygromycin (asterisk). Three biological replicates, each of five calluses, with two experimental replicates, were analyzed at each stage. The *ef1*α reference gene (M values <0.5 in these samples) was used for normalization. Dark to light color scale represents high to low expression level. All values were in the 90 to 0.01-fold range. nd: not determined. Striped: underestimated values due to callus samples containing a mixture of transgenic and non-transgenic cells.

Additionally, their expression levels were determined during *A. tumefaciens*-mediated transformation and selection using the hygromycin resistance phenotype (Figure 3). Senia wild type seeds were induced to generate primary callus and the standard transformation procedure was followed, using *A. tumefaciens* carrying pCAMBIA1300 (with the *hpt*II selection gene) to produce transgenic plants. *A. tumefaciens* not transformed with the binary plasmid was used as control, without hygromycin as a selection agent. Mature and imbibed embryos from untransformed and transgenic S-hptII plants were also analyzed. *Os.hsp18* was clearly expressed at higher levels than *Os.hsp82* in all tested tissues and stages. Expression was up to 28-fold that of the reference gene in transformed callus incubated for two weeks in hygromycin selection medium and that of mature embryos, and up to 15-fold that of *ef1α* upon *A. tumefaciens* infection. Seed imbibition rapidly resulted in mRNA decline. *Os.hsp82* had a similar expression pattern, although the level was less than that of *ef1*α in virtually all tested samples. As a control, we measured the mRNA levels of the *hptII* transgene, regulated by the CaMV 35S strong constitutive promoter. They were substantially higher than those of *Os.hsp18*, and especially *Os.hsp82*, at all stages in which there were uniquely transgenic cells (i.e. after hygromycin selection).

### *Os.hsp18* and *Os.hsp82* promoter fragments regulate the expression of transgenes in response to heat treatment

The sequences corresponding to the promoters of *Os.hsp18* and *Os.hsp82* were retrieved from the Plant Promoter Database, and 553 and 1,016 bp promoter fragments of *Os.hsp18* and *Os.hsp82*, respectively, were PCR amplified from rice var. Senia genomic DNA. Their 3′ end was at position −1 relative to the ATG translation start codon. *Os.hsp18* and *Os.hsp82* promoter fragments, pHsp18 and pHsp82, were cloned and sequence verified. The activity of these promoter fragments was experimentally assessed using the *Discosoma* spp. red fluorescent reporter protein, DsRed [26], and the epitope tag54 sequence to detect recombinant proteins through the specific mAb54 antibody [27]. The sequence encoding the DsRed-tag54-KDEL reporter (hereafter, DsRed-tag54) was placed in-frame with the sequence encoding the signal peptide of the murine monoclonal antibody mAb24 under the control of the *Os.hsp18*, *Os.hsp82* or *Zm.ubi* [32] promoters and *nos* terminator. All constructs were sequence verified, introduced in *A. tumefaciens* and used to transform rice plants, along with the *hpt*II marker for hygromycin selection. All constructs yielded hygromycin-resistant plants and those derived from different calluses were considered independent events.

**Figure 4:**
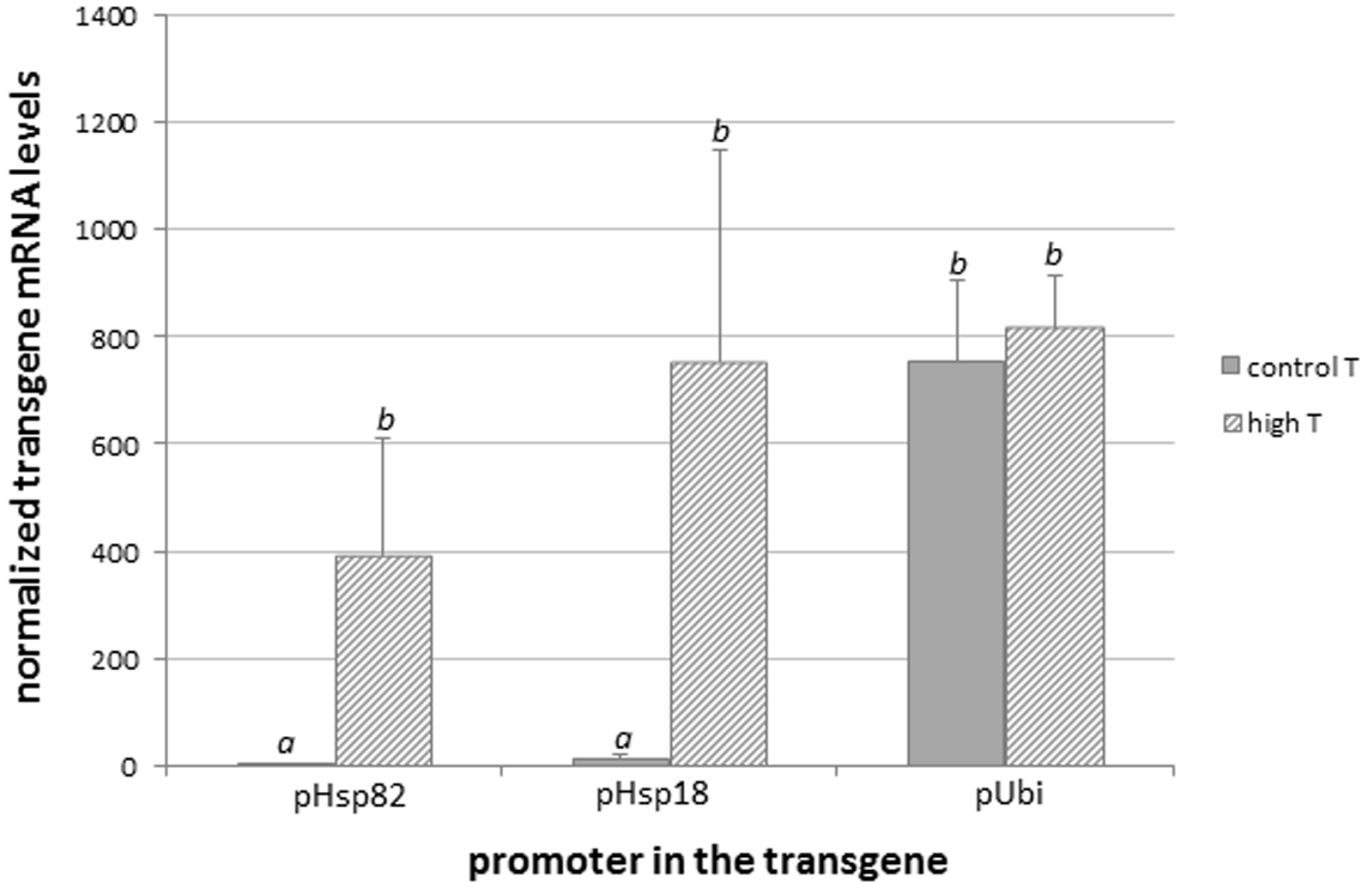
Transgene mRNA expression levels in leaves of GM rice plants in which the reporter *dsred-tag54* was regulated by three different promoters, pHsp82, pHsp18 and pUbi. Three independent events were analyzed carrying every construct. For each event, T_1_ seeds were germinated under control conditions and sets of eight plants at the V2 stage were either subjected to 42°C for 2 h, or not, and their leaves were individually sampled. Sets of five plants harboring the transgene (i.e. giving positive transgene signal using the Phire Plant kit) were collectively analyzed by RT-qPCR targeting the sequence encoding DsRed-tag54, and normalized against *ef1α*. Mean and SD values corresponding to each promoter are shown. Letters indicate statistically different transgene mRNA values (One-way ANOVA, Tukey's b posttest α<0.05).

The transgene copy number and expression profile were assessed in three independent events representing each promoter. The ratio of *dsred-tag54* to the *actin* reference gene sequence was close to 0.5 (mean and SD, 0.63±0.27), as determined by qPCR using leaf genomic DNA from T_0_ plants, suggesting single-copy insertions. Transgene expression was quantified by RT-qPCR in leaf samples of transgenic T_1_ plants at the two-leaf vegetative stage (V2), with or without exposure to 42°C for 2 h (Figure 4). Significant amounts of *dsred-tag54* mRNA were only observed when plants were kept under control temperature conditions, where the transgene was driven by the constitutive pUbi promoter. The expression values, normalized with the *ef1α* constitutive gene, were 0.47 to 2.5, 0.73 to 18.84 and 603 to 904 for pHsp82, pHsp18 and pUbi, respectively. One-way ANOVA, *P* = 0.000, gave two groups in Tukey's b posttest with α<0.05. Upon heat treatment, the pHsp18 promoter reached transgene mRNA levels from 214 to 1,117-fold that of *ef1α*, similar to those reached with the pUbi promoter. The levels of expression of the transgene was slightly lower with the pHsp82 promoter, 208 to 636-fold that of *ef1α*, but statistically similar (one-way ANOVA *P* = 0.279). As expected, no mRNA encoding DsRed-tag54 was detected in untransformed rice plants. This proved that, after exposure to heat stress, the pHsp82, and especially the pHsp18 promoter fragments, had the capacity to drive the expression of a reporter gene to levels similar to those achieved by a frequently used strong constitutive promoter.

### Use of *Os.hsp18* and *Os.hsp82* promoters to drive the expression of phytotoxic BP100 derivatives in rice

The capacity of the pHsp18 and pHsp82 promoter fragments to drive the expression of phytotoxic cationic α-helical antimicrobial peptides in stably transgenic plants was evaluated using BP100.2. The antimicrobial peptide has high phytotoxic and hemolytic activities and its constitutive expression in transgenic rice is incompatible with the survival of GM plantlets [7]. The sequence encoding BP100.2 was placed in-frame with the sequence encoding the signal peptide of the *N. tabacum* pathogenesis related protein PR1a, under the control of the pHsp18 and pHsp82 promoters. After sequence verification, the constructs were introduced in *A. tumefaciens* and used to transform rice plants along with the *hpt*II marker. The empty vector, with only the *hptII* selection gene, was transformed in parallel. Joint analysis of the transformation efficiencies achieved for constructs encoding DsRed-tag54 and BP100.2, under the control of the pHsp18, pHsp82 and pUbi promoters, showed that four out of five constructs yielded hygromycin-resistant plants with efficiencies similar to that of the empty vector (mean efficiency and SD, 97±18%, Table S3). As expected, this included all vectors encoding the non-phytotoxic DsRed-tag54 reporter, irrespective of the specific promoter regulating its expression (constitutive pUbi or heat-shock inducible pHsp18 and pHsp82).

Remarkably, the vector encoding BP100.2 under the control of the high temperature inducible promoter pHsp82 produced fertile transgenic rice plants with the same efficiency. The presence of the *bp100.2* or *dsred-tag54* transgene was confirmed in all T_0_ events using qPCR. Conversely, no GM plants were obtained with the construct encoding the highly phytotoxic BP100.2 antimicrobial peptide under the control of the pHsp18 promoter (transformation efficiency <3%). The efficiency of this transformation was at least 30-fold below that of the control plasmid, which can be considered not workable. This is in agreement with the higher activity of the pHsp18 promoter, compared to pHsp82, during transformation.

**Figure 5:**
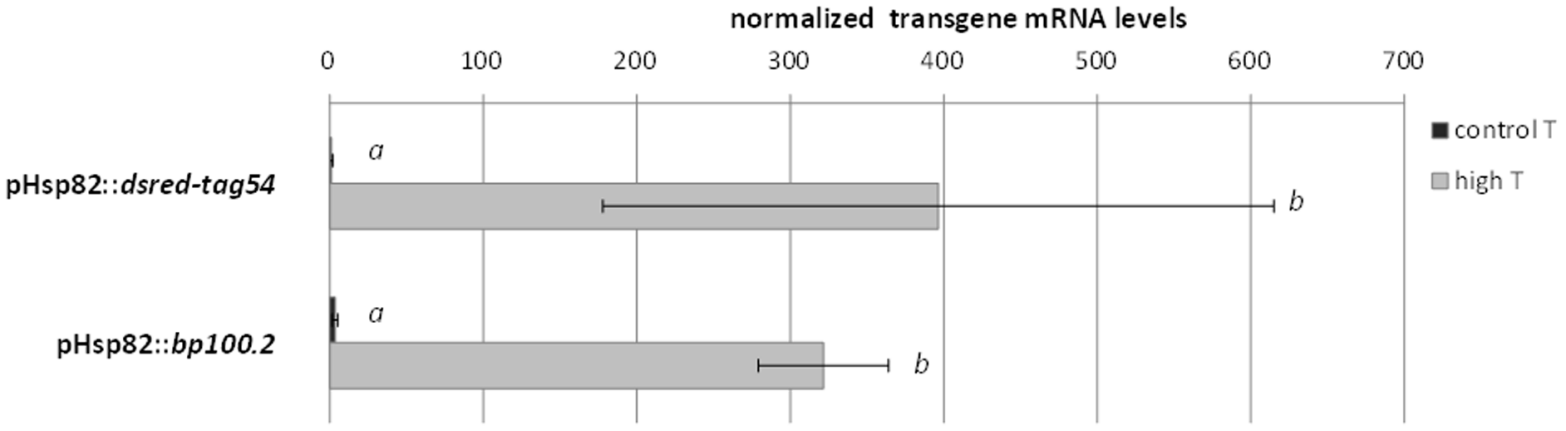
Transgene mRNA expression levels in leaves of GM rice plants harboring pHsp82::*bp100.2* or pHsp82::*dsred-tag54* constructs. Three independent events of each construct were analyzed. For each event, T_1_ seeds were germinated under control conditions and sets of eight plants at the V2 stage were either subjected to 42°C for 2 h (black bars) or not (grey bars), and their leaves individually sampled. Sets of five plants harboring the transgene (i.e. giving positive transgene signal using the Phire Plant kit) were collectively analyzed by RT-qPCR targeting the sequence encoding DsRed-tag54 or BP100.2, and normalized against *ef1*α. Mean and SD values corresponding to every transgene are shown. Different letters indicate statistically different transgene mRNA values (One-way ANOVA, Tukey's b posttest α<0.05).

Transgene expression profiles were assessed in three independent events carrying sequences encoding BP100.2 under the control of pHsp82 (pHsp82::*bp100.2*), using transgenic plants encoding DsRed-tag54 regulated by the same promoter (pHsp82::*dsred-tag54*) as the control. Hygromycin-resistant plants derived from different calluses were considered independent events. The ratio of *bp100.2* to the reference gene sequence was close to 0.5 in these events (mean and SD, 0.53±0.29) as determined by qPCR using leaf genomic DNA from T_0_ plants, suggesting single-copy insertions. Transcript levels of the *bp100.2* transgene were, prior to heat shock, 2.18 to 5.70-fold the level of *ef1α* (Figure 5), i.e. similar to those of *dsred-tag54* (One-way ANOVA *P* = 0.161). Upon heat shock, pHsp82 drove transcription to similar levels for the phytotoxic *bp100.2* (275 to 357-fold the level of *ef1α*) and the control *dsred-tag54* (One-way ANOVA *P* = 0.592). Thus, the pHsp82 promoter is a suitable tool to achieve transcription of transgenes encoding phytotoxic recombinant peptides in stably transformed GM plants.

**Figure 6:**
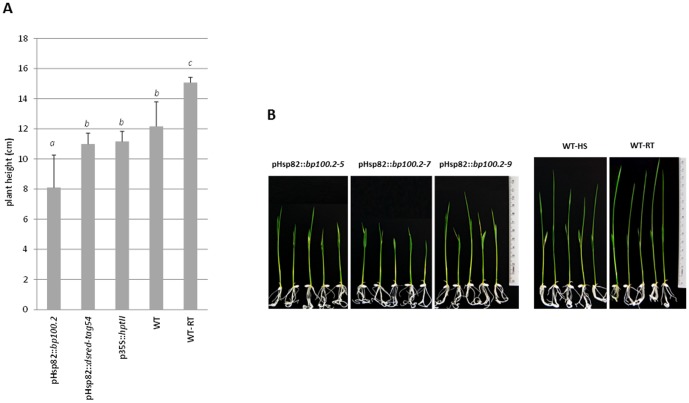
Growth of transgenic rice plants carrying pHsp82::*bp100.2* after heat shock. T_1_ seeds of rice plants carrying pHsp82::*bp100.2*, pHsp82::*dsred-tag54* (three independent events per construct) and p35S::*hptII* were germinated for 1 day in the presence of hygromycin, and five plants per event were then *in vitro* cultured, without the selection agent, up to the V2 stage (height, 6±1 cm). Five untransformed Senia plants were grown in parallel (WT). They were all treated at 42°C for 2 h and the length of their aerial parts was measured after 24 h incubation under standard conditions. Five untransformed plants were not subjected to heat stress, as an additional control (WT-RT). (**A**) Means and SD of the height of the different groups are shown. Letters indicate statistically significant differences (One-way ANOVA, Tukey b posttest with α value <0.05). (**B**) The phenotypic effects of heat treatment on transgenic plants carrying pHsp82::*bp100.2* (three independent events) and untransformed plants (WT-HS) are shown. WT-RT is shown as control.

Recombinant BP100.2 is difficult to detect due to its low extinction coefficient (low content of aromatic amino acids) and lack of immunogenicity [8]. We used its phytotoxic character to indirectly confirm the synthesis of BP100.2 in GM plants in response to heat treatment. We predicted that, in the presence of the phytotoxic recombinant BP100.2, transgenic plants would show altered phenotype after heat shock as compared to control plants. As shown in Figure 6, untransformed plants not subjected to heat treatment were taller than any temperature-stressed groups. Twenty-four hours after heat shock, plants expressing *bp100.2* were shorter than those either untransformed or expressing *dsred-tag54* or *hptII* (One-way ANOVA P = 0.000, three groups after Tukey b posttest with α<0.05) and had more serious symptoms of leaf wilting. This strongly suggested that phytotoxic recombinant BP100.2 was produced in these plants.

### Production of recombinant reporter proteins in GM rice plants using high temperature inducible promoters

Phytotoxic recombinant BP100 derivatives in transgenic plants were quantified by fusion of BP100 to a reporter moiety encompassing the fluorescent DsRed and the tag54 epitope. A construct was obtained in which the pHsp82 promoter drove the expression of BP100-DsRed-tag54-KDEL (hereafter, BP100-DsRed-tag54), with the same signal peptide and regulatory elements as the pHsp82::*bp100.2* construct. It was sequence verified and used to transform rice plants using the same *Agrobacterium*-based strategy, achieving similar transformation efficiency as the control (109%). Synthesis of *bp100-dsred-tag54* mRNA was assessed by RT-qPCR in three independent events with single copy insertions (as determined by qPCR of T_0_ leaves using *ef1α* as reference, 0.52±0.15). Residual mRNA levels in leaves of V2 plants grown under control temperature were in the range of 0.07 to 0.3-fold actin mRNA levels, but increased to 142 to 275-fold after a 2 h exposure to heat shock. These values were not statistically distinguishable from those of pHsp82-regulated transgenes expressing *bp100.2* and *dsred-tag54* in the same conditions (One-way ANOVA, *P* = 0.524 and *P* = 0.205 for control and heat treated plants, respectively).

**Figure 7:**
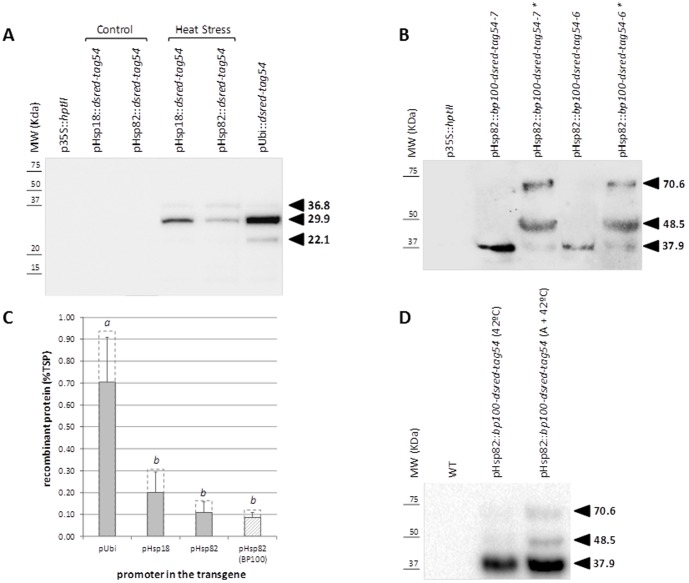
Recombinant DsRed-tag54 and BP100-DsRed-tag54 accumulation in transgenic rice seedlings. Western blot analysis of proteins from GM plants carrying: (**A**) p35S::*hptII*, pUbi::*dsred-tag54*, pHsp18::*dsred-tag54* and pHsp82::*dsred-tag54*, either treated at 42°C (heat shock) or not (control); (**B**) pHsp82::*bp100*-*dsred-tag54* (two independent events are shown) treated at 42°C. Recombinant protein in TSP was extracted from rice seedlings (five plants per event) and 20 µg of TSP per lane was boiled for five minutes and separated by SDS-PAGE before transfer to nitrocellulose filters. In lanes marked with an asterisk, insoluble pHsp82::*bp100*-*dsred-tag54* pellets were re-extracted in 8 M urea and 1% SDS buffer. Recombinant proteins were detected using the mAb54k antibody (diluted 1∶1,500) and the horseradish peroxidase-labeled anti-mouse IgG secondary antibody (diluted 1∶10,000) followed by ECL chemiluminescent detection. Five to 20 pmol of chemically synthesized controltag54 mixed with 20 µg TSP from wild type rice was used as standards for quantification. GM rice carrying p35S::*hptII* was used as control. (**C**) Recombinant protein accumulation values. Means and SD of three independent events per construct are shown in filled boxes; letters indicate statistically different values; values corresponding to the highest producer event are shown in dashed boxes. (**D**) Western blot analysis of proteins from GM plants of a single event carrying pHsp82::*dsred-tag54*, either directly subjected to 42°C or with a 6 h initial step of gradual temperature increase up to 42°C (A+42°C) (five plants per condition). Recombinant proteins were extracted in 8 M urea and 1% SDS buffer. Senia (WT) was used as control.

Transgenic plants from three independent events carrying pHsp82::*bp100-dsred-tag54* were grown to the V2 stage under controlled conditions, heat-induced for 2 h at 42°C and allowed to accumulate recombinant BP100-DsRed-tag54 for an additional two days. Three events of transgenic plants expressing *dsred-tag54* under the regulation of the pHsp82 and pHsp18 promoters were used as control. Plants with the constitutive pUbi promoter driving the expression of the same recombinant protein were included in the assay. As shown in Figure 7A, TSP extracts from all transgenic plants expressing *dsred-tag54* produced a band of 29.9 kDa in western blot assays, which is similar to the DsRed-tag54 anticipated size, and a band of about 36.8 kDa. There was an additional secondary band of 22.1 kDa that probably corresponds to a cleavage product originated by boiling TSP before SDS-PAGE [33]. TSP extracted from all three samples expressing *bp100-dsred-tag54* under the control of pHsp82 produced a band of an apparent MW of 37.9 kDa (Figure 7B), which is in agreement with the 36.8 kDa band detectable in extracts of plants producing recombinant DsRed-tag54. When higher amounts of protein extracts were analyzed, a faint band of the expected size (31.5 kDa) was also visible (Figure S2). The BP100 portion of the recombinant molecule seemed to strongly enhance the proportion of the slower band, which was probably the result of incomplete denaturation or interaction of different molecules. Subsequent analysis of the insoluble protein fraction showed that a significant proportion of immune-reactive proteins, with apparent MW of 37.9, 48.5 and 70.6 kDa, could only be extracted in an SDS and urea-based, strongly denaturing buffer (Figure 7B). As expected, no immune-reactive bands were observed upon analysis of TSP from untransformed plants, those uniquely expressing the hygromycin resistance selection marker, or those transformed with pHsp82::*dsred-tag54* and not subjected to high temperature (Figure 7A). Thus, phytotoxic recombinant BP100 derivatives were produced and accumulated after heat induction in transgenic rice using the pHsp82 heat inducible promoter. Quantification of the immune-reactive bands (Figure 7C) showed that the pUbi promoter led to approximately 0.9% TSP of DsRed-tag54, while promoters derived from *Os.hsp18* and *Os.hsp82* led to recombinant DsRed-tag54 levels up to 0.3 and 0.15% TSP, respectively, after heat shock. Finally, use of the same pHsp82 promoter to drive the expression of the phytotoxic BP100 fusion protein led to similar accumulation levels, up to 0.12% TSP in these conditions.

One week after germination, pHsp82::*bp100-dsred-tag54* and control pHsp82::*dsred-tag54* transgenic plantlets from three different events per construct were induced by heat shock, and the recombinant chimeric protein allowed to accumulate and mature for three days prior to confocal microscopy of the plantlet radicles. All analyzed transgenic plants displayed red fluorescence, further confirming the accumulation of the recombinant proteins. Fluorescence intensities were measured in four fields per radicle. The p82::*bp100-dsred-tag54* and control p82::*dsred-tag54* plants expressed similar levels of recombinant proteins, with mean and SD values of 1258±420 and 981±286 fluorescence units per field, respectively (One-way ANOVA *P* = 0.135). This demonstrated that recombinant phytotoxic BP100-derived peptides could be accumulated to levels similar to those of the DsRed-tag54 reporter in stable GM plants constructed with the pHsp82 temperature inducible promoter. The DsRed-tag54 fluorescence showed a typical ER reticular pattern accompanied by a few spherical structures, attributable to the known effects of high temperatures on the internal organization of the cell, such as fragmentation of the Golgi system and the ER [34]. In contrast, fluorescence in pHsp82::*bp100-dsred-tag54* radicles was essentially in numerous and widely distributed vesicles, obscuring the ER network (Figure S3), in agreement with BP100 inducing ER-derived protein bodies [8].

## Discussion

Biotechnological production of cationic α-helical antimicrobial peptides that have potent activities against pathogenic microorganisms is of great interest [35], but their constitutive expression is not compatible with the viability and fertility of host plants [7]. This problem has been previously approached by expression of peptides (i) with suboptimal antimicrobial activity linked to lower phytotoxicity [36], and (ii) with rational sequence modification to achieve a targeted reduction in phytotoxicity without affecting antimicrobial potency [8]. Modifications often alter the properties of the peptides not only in terms of antibacterial activity but also the range of target bacterial species [3], [5], [37]. This makes the design of a peptide with optimal properties difficult [38]. A tool to produce highly active peptides of interest in plants, irrespective of their phytotoxic character, is desirable. Inducible promoters could potentially reduce the negative impact of toxic recombinant peptides on the transgenic plant by forcefully restraining the presence of the peptide during plant development, while allowing high expression upon induction. The probable harmful effects of accumulation of recombinant phytotoxic compounds after induction, to the plant biofactory, are irrelevant since the main objective is recovery of the recombinant product. We predicted that it would only be possible to successfully generate transgenic plants carrying transgenes that encoded phytotoxic peptides by using promoters extremely inactive throughout transformation and regeneration, due to the vulnerability of the plant material during this process. Due to the practicality and low cost of using high temperature as an inducing agent in plants, and the extensive literature on the plant response to heat shock [39]–[44], we focused on heat shock promoters to assess the feasibility of obtaining recombinant phytotoxic antimicrobial peptides in rice, using as model, highly active and phytotoxic peptides derived from BP100.

Profiling technologies such as microarray hybridization have been widely used to systematically investigate transcriptomic changes during plant development, in different tissues and in response to diverse environmental conditions, including heat shock [45]–[47]. We selected candidate promoters *in silico* on the unique basis of the massive transcriptome information on publically available web sites (MIAMExpress, CREP), specifically focusing on studies on prolonged heat shock transcriptional response [10]. A similar strategy has recently been used to identify abiotic stress-inducible promoters intended to drive the overexpression of genes encoding nontoxic, stress resistance proteins in transgenic plants, with the aim of reducing the stunted growth and decrease in yield that may be caused by constitutive overexpression of these transgenes [48]. In contrast, here we selected candidate promoters for the expression of phytotoxic recombinant peptides, in plants, to drive high gene expression after heat treatment with minimal gene expression under any other condition, especially focusing on callus during agrotransformation and different tissues in the germination and seedling developmental stages.

The four sequences which best fulfilled these requirements [AK063751.1 (*Os.hsp82*), X60820.1, AU165294 and AK071240 (*Os.hsp18*), Figure 1] encoded heat shock proteins belonging to the Hsp90 (*Os.hsp82*) and the small Hsp (sHsp) families (X60820.1, AU165294 and *Os.hsp18*, UniProt database). Heat shock proteins are found in every organism [49], and play important roles in cell protection against deleterious effects of stress e.g. acting as molecular chaperones [49]–[55] and in cellular functions related to growth and development [49], [56]. Small Hsps are the most abundant, heterogeneous and stress-responsive group of Hsp in higher plants [57]–[60]. The genes for more than 20 sHsps have been identified in rice, with specific spatial and temporal regulation under stress and in developmental stages [42], [61]. Specifically, the *Os.hsp18* gene, encoding the cytosolic class II Hsp18.0, had very high expression upon induction, although our microarray analysis showed that it retained some activity in developmental stages and conditions that are fundamental to the production of GM plants. Hsp90 are, in general, constitutively expressed and among the most abundant proteins in cells, although their expression increases in response to stress [39]. *Os.hsp82* had extremely low expression in control tissues and reached high levels of expression in response to heat, but *Os.hsp18* and especially *Os.hsp82* were not significantly induced by other stress conditions, such as cold, NaCl and drought, in agreement with previous publications [10], [62], [63]. Their moderate mRNA levels in seeds, as determined using the CREP platform, would most probably allow fertility of the plants. Experimental assessment of the expression patterns of these two genes in the rice genotype of interest, Senia, allowed ratification of the *Os.hsp18* and *Os.hsp82* candidate sequences [10], [62], [64]. In a thorough analysis of *A. tumefaciens*-mediated transformation of Senia rice and selection using the hygromycin resistance trait, *Os.hsp18* and *Os.hsp82* mRNA levels were consistently 20 and 60-fold lower, respectively, than in leaves under induction conditions (the highest values were a transient response to *Agrobacterium* infection and hygromycin treatment) and about 10 and 300-fold lower, respectively, than those of the *hptII* transgene regulated by the p35S constitutive promoter. We therefore considered that *Os.hsp82*, and perhaps *Os.hsp18*, promoters would be able to drive the expression of transgenes encoding phytotoxic peptides in rice, possibly the latter with higher yields, and allow survival of GM plants.

Five hundred to 1,000 base pair promoter fragments of *Os.hsp18* and *Os.hsp82* regulated the expression of a reporter sequence in leaves of transgenic rice plants, in a similar manner as the endogenous genes. The low transgene mRNA levels measured prior to heat shock in GM plants carrying pHsp18::*dsred-tag54* and pHsp82::*dsred-tag54* (about 10 and 1-fold those of the reference gene, respectively) increased to approximately 700 and 400-fold that of the reference gene 2 hours after heat treatment. Even though different GM events had different transgene mRNA levels, globally the expression of endogenous *Os.hsp18* and *Os.hsp82* genes was similar to, respectively, that of the *dsred-tag54* transgene driven by the pHsp18 and pHsp82 promoters (One-way ANOVA *P* = 0.465 and *P* = 0.463, respectively). In addition, western blot analyses and confocal microscopy showed accumulation of recombinant DsRed-tag54 specifically upon heat shock in leaves and roots carrying pHsp18::*dsred-tag54* (data not shown) and pHsp82::*dsred-tag54*. The selected pHsp18 and pHsp82 promoter fragments retained the essential elements to drive heat shock induction in these tissues, and they performed as expected in GM plants. This is in agreement with previous publications where regulatory elements including the TATA box were *in silico* predicted in the two 5′ proximal regions [65], [66].

Use of the pHsp18 promoter to drive the expression of the phytotoxic peptide BP100.2 did not allow survival of fertile transgenic plants. Remarkably, transgenic plants producing BP100.2 and BP100-DsRed-tag54, also phytotoxic [8], were obtained with pHsp82, with the same efficiency as those expressing a non-toxic recombinant protein such as the reporter DsRed-tag54. After heat treatment, leaves of plants carrying pHsp82::*bp100.2* and pHsp82::*bp100-dsred-tag54* produced levels of transgene mRNA similar to those harboring the *dsred-tag54* reporter under the control of the same pHsp82 promoter, indicating that these transgenes were fully functional. As BP100 is difficult to detect due to lack of antigenicity and a low extinction coefficient [8], a phenotypic approach was indirectly used to prove the synthesis of BP100.2 in these cells. High temperatures are known to cause wilting and a significant decline in relative growth rate [67]. Growth of pHsp82::*bp100.2* plants after a high temperature shock was slower than that of untransformed or transgenic control plants and there was considerably more wilting of their leaves, which supported the production of the phytotoxic recombinant BP100.2 peptide in these cells.

In view of the somewhat higher expression of *Os.hsp18* than *Os.hsp82* during transformation and regeneration (about 5–10 fold), the finding that pHsp82, but not pHsp18, was able to drive the expression of phytotoxic recombinant peptides in plants supports our initial hypothesis and experimental approach. It is essential that promoter sequences to regulate the expression of phytotoxic transgenes in plants are chosen on the basis of their extremely low basal activity in the tissues, the developmental stages and under the conditions for production of GM plants and germination. No viable transgenic plants were obtained that expressed BP100.2 under the control of 1000-bp promoter fragments of the *Os.37773.1.s1_at* (AU165294) and *Os.165.1.s1_at* genes, with mRNA levels above those of *Os.hsp18* during the transformation process (Figure 1). These results place the threshold of basal expression of a phytotoxic transgene during production of the GM plant at very low levels, similar to those of the *Os*.*hsp82* gene. We then re-evaluated the initially selected heat stress-responsive genes on the basis of this threshold. Five additional sequences (AB110191.1, AU165294, AK069860.1, X60820.1 and AB098712.1, Table S2) had higher expression than *Os.hsp82* after heat shock, and mRNA levels below *Os.hsp18* in control conditions. Four of them were expressed at levels above the *Os.hsp18* threshold during transformation (Figure 1), so their promoters would not be suitable for producing GM plants by driving the expression of phytotoxic components. As X60820.1 had expression patterns similar to *Os.hsp82*, its promoter most probably would be suitable to drive this expression. However, it is unlikely that yields would be substantially above those of pHsp82, as deduced from its moderate transcriptional response to heat shock.

Recombinant BP100 derivatives were produced in transgenic plants in response to heat treatment through regulation of the pHsp82 promoter. Three days after induction, BP100-DsRed-tag54 represented 0.12% of seedling proteins solubilized in urea and SDS containing buffer, determined by western blot using the tag54 epitope. Using the same promoter to drive the expression of DsRed-tag54 yielded up to 0.15% TSP, harsher denaturing conditions not significantly increasing the yield (data not shown). Although an anti-tag54 reactive band of the anticipated size was visible in TSP from heat-treated pHsp82::*bp100-dsred-tag54* leaves, the major bands, especially those only extracted under strongly denaturing conditions, had slower mobility, which is compatible with remaining BP100-driven molecular interactions. This could be explained by the unusual physicochemical properties of BP100 (highly cationic, pI = 11.5), known to result in interactions with many components of the cell and reduce the efficiency of isolation from complex matrices [8]. In agreement, after the same temperature treatment, BP100-DsRed-tag54 was accumulated at statistically similar levels to the reporter DsRed-tag54, as determined by quantification of the fluorescence signal in confocal micrographs (i.e. when no extraction is needed). We have previously described [8] how the BP100 sequence induces the formation of ER-derived vesicles or protein bodies (PB), functioning similarly to the tandemly repeated VPGXG elastin-like motif [68], hydrophobins [69] and the γ-zein derived Zera polypeptide [70]. Recombinant BP100-DsRed-tag54 accumulates in these vesicles, together with other luminal ER proteins, in transiently transformed *N. benthamiana* leaves [8]. Here we observed that recombinant BP100-DsRed-tag54 had a similar vesicular pattern in radicles of transgenic rice after high temperature induction, further establishing that recombinant BP100 derivatives accumulate in newly formed ER-derived vesicles in different host plant species.

It is therefore clear that phytotoxic compounds such as BP100 can be expressed in stably transformed plants using a strategy based on inducible promoters. Constitutive expression of this type of transgene has only previously been achieved in transient systems, the harmful effects of the recombinant proteins strongly interfering with normal growth and development of the transgenic plants. With the pHsp82 promoter, we estimate a yield of about 0.1% TSP in our initial induction conditions. Larkindale and Vierling [71] have showed that different acclimation schemes resulted in different yields of Hsp in *Arabidopsis*. Notably, a gradual temperature increase led to higher transcript levels than heat shock without acclimation. Here, use of an acclimation treatment (6 h gradual increase prior to 2 h at 42°C) increased the recombinant protein yield 3-fold (Figure 7D). Thus, this approach allowed obtaining up to about 0.3% total extracted phytotoxic recombinant proteins. This is about half the yield of the BP100.gtag peptide, specifically designed to display extremely low toxicity, achieved using the constitutive maize pUbi promoter in the same rice system [8]. Recombinant Cry1B has been produced in rice at similar yields (0.2% TSP) under the control of the maize proteinase inhibitor (Mpi) wound-inducible promoter [72]. Higher yields of recombinant *Acidothermus cellulolyticus* endoglucanase E1, 1.3% TSP, have been achieved in transgenic tobacco using the tomato Rubisco small subunit (RbcS-3C) light-inducible promoter [73]. Note that this type of highly inducible promoter does not allow production of phytotoxic compounds.

In a prospective exercise, our experimental approach was used to identify alternative promoter sequences with increased performance to drive the expression of phytotoxic peptides in plants. Transcriptome-wide datasets are available for rice seedlings under control and a range of abiotic stress conditions, such as salinity, desiccation, suboptimal temperature (4°C), and various minerals, elicitor and hormone treatments, and were analyzed according to the above mentioned criteria. A total of 23 sequences were identified with high expression under induction conditions (above that of *Os.hsp82*) and minimal expression levels in control conditions (below that of *Os.hsp18*) (Figure S4). On assessment of their expression patterns in callus and during the transformation process and production of transgenic plants (taken as the mean of the microarray normalized data in these tissues), there were only four sequences with mRNA levels below *Os.hsp82*, strongly suggesting that their promoters would serve to drive the expression of phytotoxic proteins in transgenic plants. They were induced by drought, NaCl, phosphorus and the heavy metals chromium (VI) and arsenic. A single sequence, Os.49245.1.S1_at, reached very high levels after drought and NaCl induction (similar to *Os.hsp18* after heat shock), making its promoter the best candidate. Five additional sequences had mRNA levels below *Os.hsp18* but above *Os.hsp82* in the analyzed tissues and developmental stages. Their promoters might also be suitable to drive the expression of toxic compounds in GM plants, but their expression levels after induction were below that of *Os.hsp18*. The best use of our promoter-selection strategy would most likely be achieved by using longer-term expression data to select sequences with the longest span of transcriptional response to the stimulus, which we could speculate would result in higher accumulation of the recombinant proteins.

In conclusion, we produced phytotoxic α-helical antimicrobial peptides derived from BP100 in plants on the basis of strict regulation of transgene transcription. A requirement was that transcription was below a rigorous threshold in specific plant tissues and developmental stages, particularly during transformation and regeneration of GM plants. The heat shock induced promoter, pHsp82, fulfilled this condition and was capable of driving production of phytotoxic recombinant peptides in plants, although the yield is rather low for most commercial applications. In addition, we demonstrate that, thanks to the increasing information available, *in silico* analysis of transcriptome profiles is a suitable and inexpensive approach to select promoters with specific activity patterns.

## Supporting Information

Figure S1 **A summary of the expression profiles of 16 genes selected as having the highest expression or induction in response to heat-shock in 7-day IR64 (** ***indica*** **) seedlings subjected to cold, salt and drought stress for 3 h.** Data on 14-day ZhongHua 11 (*japonica*) seedlings subjected to heat shock for 3 h are also shown. Dark to light color scale represents high to low expression levels, black corresponding to 11,309 normalized fluorescence units. Arrows indicate the two sequences whose promoters were selected to produce transgenic plants.
(TIF)

Figure S2 **Recombinant BP100-DsRed-tag54 accumulation in transgenic rice seedlings.** Western blot analysis of proteins from GM plants carrying pHsp82::*dsred-tag54* and pHsp82::*bp100-dsred-tag54* (two independent events taken as example) treated at 42°C. Recombinant protein in TSP was extracted from rice seedlings (five plants per event) and 30 µg of TSP per lane was boiled for five minutes and separated by SDS-PAGE before transfer to nitrocellulose filters. Recombinant proteins were detected using the mAb54k antibody (diluted 1∶1,500) and the horseradish peroxidase-labeled anti-mouse IgG secondary antibody (diluted 1∶10,000) followed by ECL chemiluminescent detection.
(TIF)

Figure S3 **Confocal micrographs of rice radicles from transgenic plantlets carrying pHsp82::** ***bp100-dsred-tag54*** **(A and C) and control pHsp82::** ***dsred-tag54*** **(B and D), at the V2 developmental stage, subjected to 42°C for 2 hours and further incubated under control growth conditions for three days in a culture chamber.** (A and B), DsRed fluorescence; (C and D), bright field. Scale bars: 0.5 µm.
(TIF)

Figure S4 **A summary of the expression profiles of a selection of sequences induced by temperature, drought and NaCl stress, and hormone treatment, in callus and seedlings of rice, based on the following** ***in silico*** **published data [74]–[79]** **.** Genes with mRNA levels below that of *Os.hsp18* in control seedlings (control) and above that of *Os.hsp82* after induction (induced) are shown. Normalized expression levels in 28 tissues, relevant to the process of obtaining transgenic plants, of three rice genotypes: Minghui 63 (M, *indica*), Zhenshan 97 (Z, *indica*) and Nipponbare (N, *japonica*) (grey scale). The mRNA levels in these tissues and developmental stages were estimated using the mean expression values (green scale). Dark to light color scale represents high to low expression levels, the darkest corresponding to 37,405 (grey scale) and 12,480 (green scale) normalized fluorescence units.
(TIF)

Table S1 **Primer sequences and PCR conditions used.** Additional restriction sites are underlined.
(DOCX)

Table S2 **Sequences with the greatest expression changes in response to heat stress in rice, extracted from publically available microarray hybridization data.** Details on the sequence (representative public ID, Affymetrix code and description) and the mRNA expression in response to treatment at 42°C for 3 h [normalized fluorescence units in rice seedlings under control (control) and heat-shock (heat-shock) conditions; fold change (fold) and difference (HS-C) of normalized fluorescence intensities in the two conditions are also indicated]. Note that the sequences with the same Affymetrix number correspond to the same gene. Dark to light scale of shading represents high to low expression level. The promoters of the two sequences indicated in bold were selected to produce transgenic plants.
(DOCX)

Table S3 **Transformation efficiencies of the different constructs encoding the DsRed-tag54 reporter or the phytotoxic BP100.2 AMP, under the control of a rice heat-shock (pHsp18 and pHsp82) or a maize constitutive (pUbi) promoter.**
(DOCX)
