# Supplementary figures and images for: Production of Phytotoxic Cationic α-Helical Antimicrobial Peptides in Plant Cells Using Inducible Promoters
Source: PLoS One. 2014 Nov 11;9(11):e109990. doi: 10.1371/journal.pone.0109990 (PMC4227650; doi:10.1371/journal.pone.0109990)

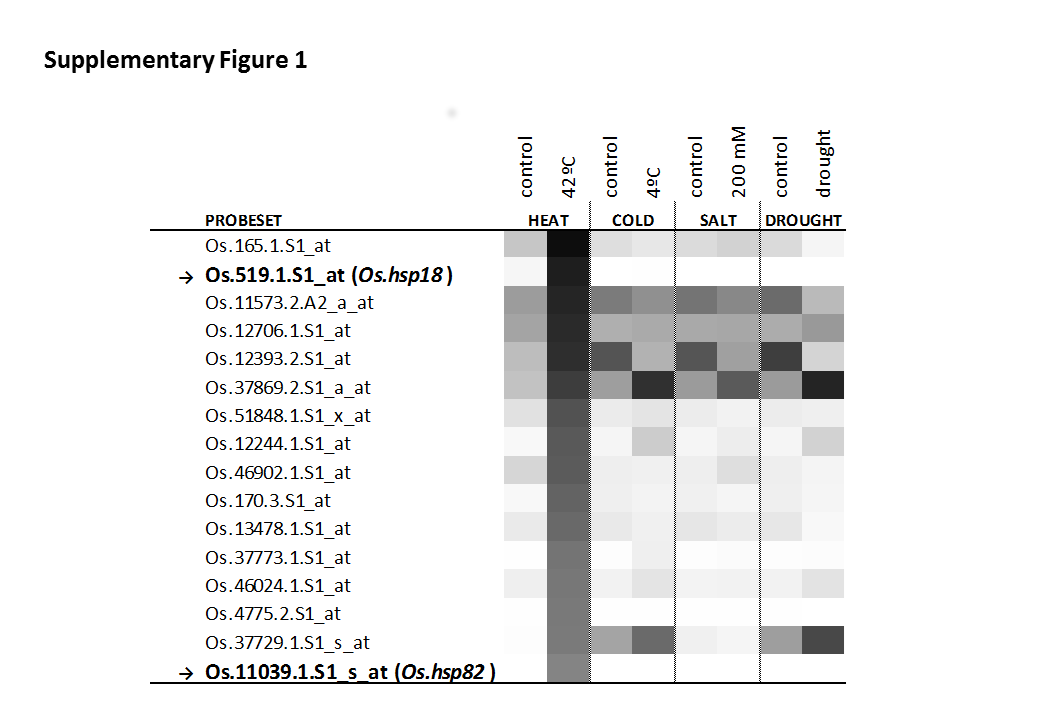

Supplement: Figure S1 — A summary of the expression profiles of 16 genes selected as having the highest expression or induction in response to heat-shock in 7-day IR64 ( indica ) seedlings subjected to cold, salt and drought stress for 3 h. Data on 14-day ZhongHua 11 (japonica) seedlings subjected to heat shock for 3 h are also shown. Dark to light color scale represents high to low expression levels, black corresponding to 11,309 normalized fluorescence units. Arrows indicate the two sequences whose promoters were selected to produce transgenic plants. (TIF) [file pone.0109990.s001.tif]

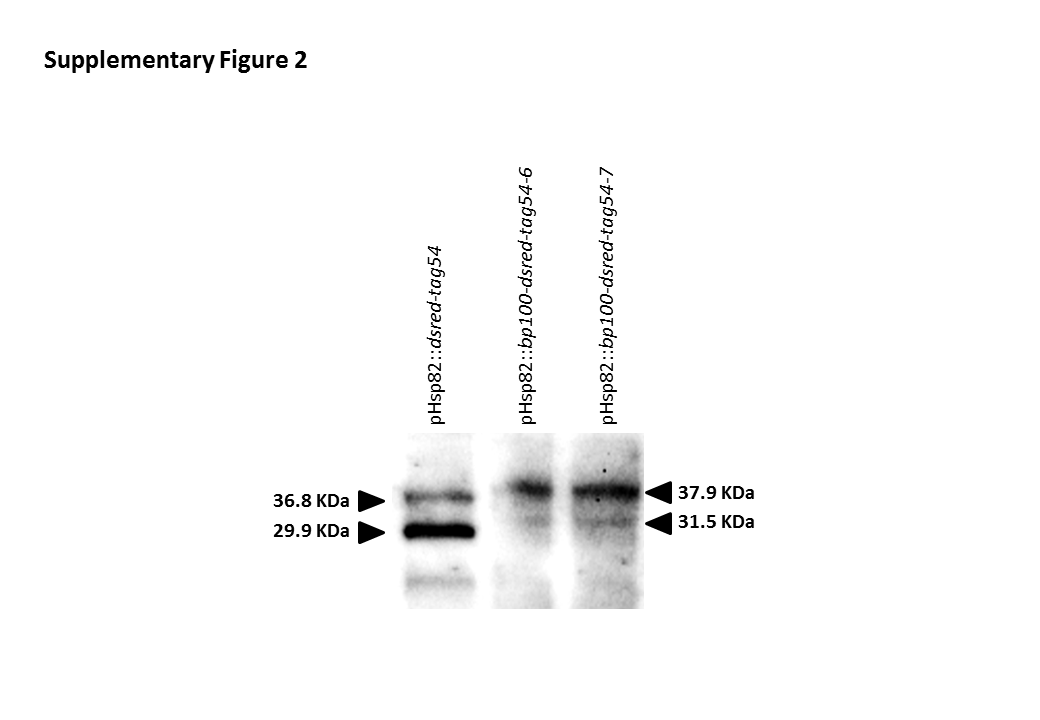

Supplement: Figure S2 — Recombinant BP100-DsRed-tag54 accumulation in transgenic rice seedlings. Western blot analysis of proteins from GM plants carrying pHsp82::dsred-tag54 and pHsp82::bp100-dsred-tag54 (two independent events taken as example) treated at 42°C. Recombinant protein in TSP was extracted from rice seedlings (five plants per event) and 30 µg of TSP per lane was boiled for five minutes and separated by SDS-PAGE before transfer to nitrocellulose filters. Recombinant proteins were detected using the mAb54k antibody (diluted 1∶1,500) and the horseradish peroxidase-labeled anti-mouse IgG secondary antibody (diluted 1∶10,000) followed by ECL chemiluminescent detection. (TIF) [file pone.0109990.s002.tif]

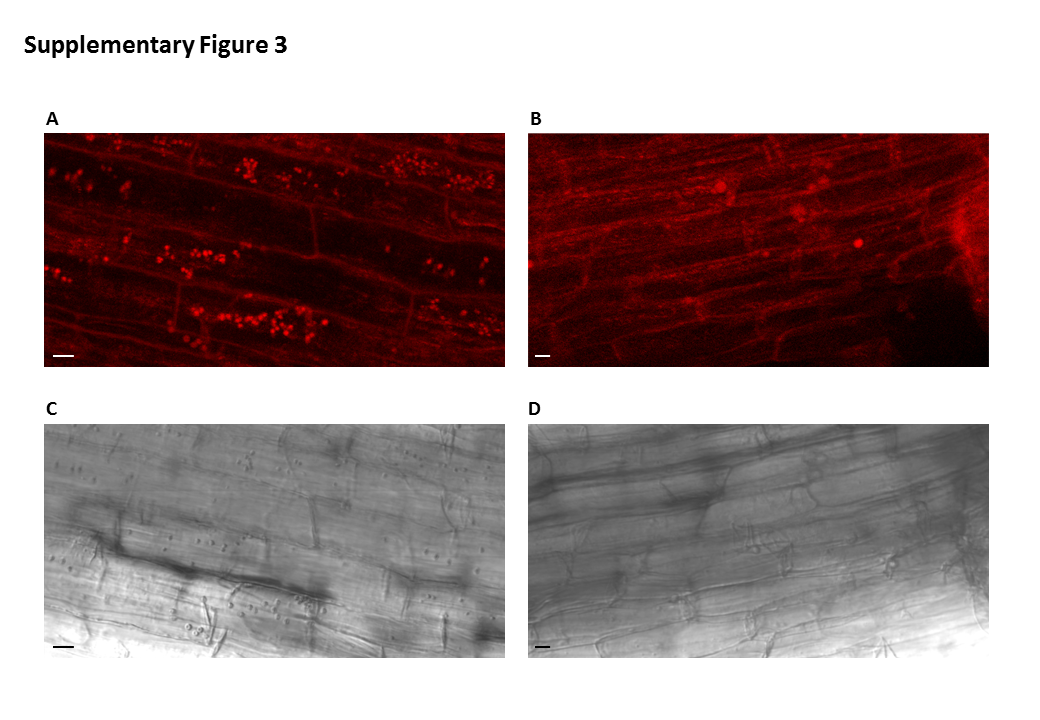

Supplement: Figure S3 — Confocal micrographs of rice radicles from transgenic plantlets carrying pHsp82:: bp100-dsred-tag54 (A and C) and control pHsp82:: dsred-tag54 (B and D), at the V2 developmental stage, subjected to 42°C for 2 hours and further incubated under control growth conditions for three days in a culture chamber. (A and B), DsRed fluorescence; (C and D), bright field. Scale bars: 0.5 µm. (TIF) [file pone.0109990.s003.tif]

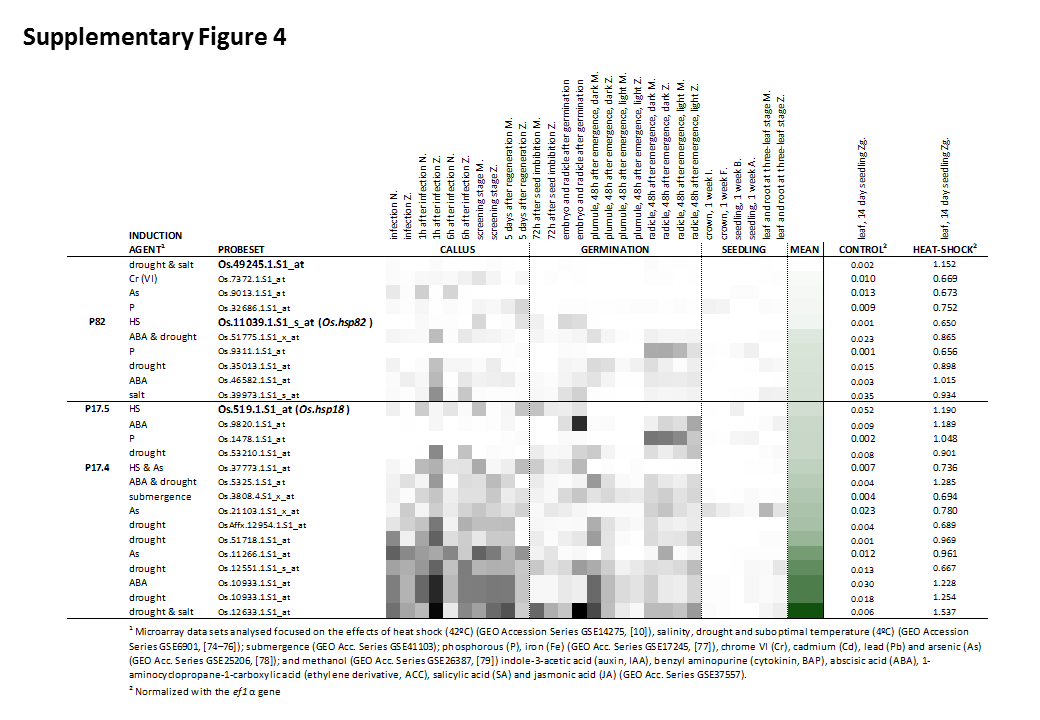

Supplement: Figure S4 — A summary of the expression profiles of a selection of sequences induced by temperature, drought and NaCl stress, and hormone treatment, in callus and seedlings of rice, based on the following in silico published data [74]–[79] . Genes with mRNA levels below that of Os.hsp18 in control seedlings (control) and above that of Os.hsp82 after induction (induced) are shown. Normalized expression levels in 28 tissues, relevant to the process of obtaining transgenic plants, of three rice genotypes: Minghui 63 (M, indica), Zhenshan 97 (Z, indica) and Nipponbare (N, japonica) (grey scale). The mRNA levels in these tissues and developmental stages were estimated using the mean expression values (green scale). Dark to light color scale represents high to low expression levels, the darkest corresponding to 37,405 (grey scale) and 12,480 (green scale) normalized fluorescence units. (TIF) [file pone.0109990.s004.tif]
