# Supplementary material for: Production of Phytotoxic Cationic α-Helical Antimicrobial Peptides in Plant Cells Using Inducible Promoters
Source: PLoS One. 2014 Nov 11;9(11):e109990. doi: 10.1371/journal.pone.0109990 (PMC4227650; doi:10.1371/journal.pone.0109990)
Supplement: Table S1 — Primer sequences and PCR conditions used. Additional restriction sites are underlined. (DOCX) [file pone.0109990.s005.docx]

**Supplementary Table 1**

| Target sequence | Primer | Primer sequence | MgCl_2_ (mM) |  | Primer conc. (nM) | Annealing temperature (ºC) |
| --- | --- | --- | --- | --- | --- | --- |
| ***Os.hsp18 promoter*** | pHsp18_Kpn | TATAGGTACCAGGATATAAATTAACAGCGC | 3 |  | 500 | 49 / 59 |
|  | pHsp18_Spe | GTAGACTAGTCTCTCGATCGCCTCTTC |  |  | 500 |  |
| ***Os.hsp82 promoter*** | pHsp82_Kpn | TATAGGTACCATACGTTTATTAGCAATAATAGTTT | 3 |  | 500 | 50 / 62 |
|  | pHsp82_Spe | GTAGACTAGTGTTGATCTCTGCCTGGAA |  |  | 500 |  |
| ***bp100.2 coding sequence*** | PR1a_Spe | TATAACTAGTGAGGCCACCATGGG | 1.5 |  | 300 | 49 / 58 |
|  | BP100KDEL_Bam | TATAAAGCTTGGATCCATTATCAGAGCTC |  |  | 300 |  |
| ***bp100-dsred-tag54 and***  ***dsred-tag54 coding sequence*** | CHS_Spe | TATAACTAGTGAATTCACAACACAAATCAGA | 1.5 |  | 300 | 49 / 58 |
|  | TagKDEL_Bam | TATAGGATCCTACTAAAGCTCATCCTTCTCA |  |  | 300 |  |
| ***β-actin rice gene*** | ACTrice_for* | CCTCTTCCAGCCTTCCTTCATA |  |  | 100 | 60 |
|  | ACTrice_rev* | GCAATGCCAGGGAACATAGTG |  |  | 100 |  |
| ***ef-1a rice gene*** | EFrice_for* | TTTCACTCTTGGTGTGAAGCAGAT |  |  | 300 | 60 |
|  | EFrice_rev* | GACTTCCTTCACGATTTCATCGTAA |  |  | 300 |  |
| ***bp100.2 transgene*** | bp100der_for* | TCCTCGTGATCTCCCACTCCTG |  |  | 100 | 60 |
|  | bp100der_rev* | CGGATCCATTATCAGAGCTCGT |  |  | 50 |  |
| ***dsred transgene*** | SYDsRed_for | TGGCAAAGAAGCCTGTGCAGC |  |  | 100 | 60 |
|  | SYDsRed_rev | TGGTGGCGTCCCTCGGTTCT |  |  | 100 |  |
| ***Os.hsp18 rice gene*** | P17,5SYR_for | AACGCAGACGTCGACAAGAT |  |  | 100 | 60 |
|  | P17,5SYR_rev | CGACGCAGATGCAGAGAGAT |  |  | 100 |  |
| ***Os.hsp82 rice gene*** | P82SYR_for | TCGACGACCCAAACACCTTC |  |  | 100 | 60 |
|  | P82SYR_rev | CATTTCGACATGGCAGCGAG |  |  | 100 |  |
| ***T_1_ leaf material (dsred)*** | DsRed_for | GTCATGAACTTTGAAGACG | 1.5 |  | 500 | 55 |
|  | Nos.te_rev | TAATCATCGCAAGACCGGCA |  |  | 500 |  |
| ***T_1_ leaf material (bp100.2)*** | P82_for | ATCGGAGCAGTGAACTGA | 1.5 |  | 500 | 57 |
|  | Nos.te_rev | TAATCATCGCAAGACCGGCA |  |  | 500 |  |

*[7]
