# Supplementary material for: Production of Phytotoxic Cationic α-Helical Antimicrobial Peptides in Plant Cells Using Inducible Promoters
Source: PLoS One. 2014 Nov 11;9(11):e109990. doi: 10.1371/journal.pone.0109990 (PMC4227650; doi:10.1371/journal.pone.0109990)
Supplement: Table S2 — Sequences with the greatest expression changes in response to heat stress in rice, extracted from publically available microarray hybridization data. Details on the sequence (representative public ID, Affymetrix code and description) and the mRNA expression in response to treatment at 42°C for 3 h [normalized fluorescence units in rice seedlings under control (control) and heat-shock (heat-shock) conditions; fold change (fold) and difference (HS-C) of normalized fluorescence intensities in the two conditions are also indicated]. Note that the sequences with the same Affymetrix number correspond to the same gene. Dark to light scale of shading represents high to low expression level. The promoters of the two sequences indicated in bold were selected to produce transgenic plants. (DOCX) [file pone.0109990.s006.docx]

**Supplementary Table 2**
